# Biology must develop herd immunity against bad-actor molecules

**DOI:** 10.1371/journal.ppat.1007038

**Published:** 2018-06-28

**Authors:** Richard K. Plemper, Robert M. Cox

**Affiliations:** Institute for Biomedical Sciences, Georgia State University, Atlanta, Georgia, United States of America; University of Michigan Medical School, UNITED STATES

In 2016, the 10 most prescribed therapeutics in the United States were again small-molecule drugs [1]. Small molecules have been game changing for infectious disease prevention and case management. However, novel antimicrobials are urgently needed to address emerging drug resistance, improve tolerability, and pioneer therapeutic options against currently untreatable infections. Drug discovery was originally the domain mostly of the pharmaceutical industry, but academic researchers have increasingly entered the field.

## The issue

While these efforts have yielded viable lead candidates, they have also been compromised by the (re)discovery of undesirable “bad actor” compounds in vast numbers. In the medicinal chemistry field, bad actors are small-molecule screening hits that supposedly show a specific bioactivity but are undevelopable and/or lack true selectivity for the proposed target. Four different classes of these undesirable hits emerge frequently: i) compounds directly blocking assay signals [2]; ii) unreproducible hits due to impurities or decomposition of the matter [3]; iii) self-aggregating compounds that nonspecifically absorb targets [4]; and iv) frequently covalently reactive pan-assay interference compounds (PAINS) [5, 6] and their natural product companions, invalid metabolic panaceas (IMPs) [7].

Whereas class i to iii problem hits can often be identified through orthogonal counterscreens and variation of experimental conditions [8], PAINS and IMPs can be difficult to spot. They were first noted based on their frequent appearance in independent high-throughput–screening (HTS) campaigns. Their initial bioactivity profiles are typically attractive and often extend to counterscreens, making triaging challenging. In contrast to well-behaved hits, however, PAINS and IMPs generate a variety of misleading assay results originating, amongst others, from covalent, unspecific protein reactivity [9], redox activity [10], and membrane-interference–disturbing cellular pathways [11]. Synthetic hit-to-lead development efforts of these chemotypes proved largely unsuccessful, consuming resources without a meaningful return.

The most relevant outcome of these efforts was the recognition of the problem by the medicinal chemistry community and the short listing of chemotypes that should best be discarded or, if selected for development, be advanced with great scrutiny [8]. A groundbreaking first PAINS compendium comprised over 450 structural classes [6], and several cheminformatics filters were subsequently developed to flag supposedly compromised substructures (Table 1). While this initiative has enjoyed much support from medicinal chemists [12, 13], recent studies have cautioned against an uncritical application of electronic filters, since a broad assessment of HTS data sets suggested that many chemical scaffolds may be unduly flagged, while some high-frequency–hit compounds pass undetected [14–16]. This debate in the medicinal chemistry community reflects that these early algorithms were derived from screens using a single approximately 100,000-entry library and assay type [6], which creates some condition-specific bias of the filters [10]. However, it was rightly pointed out that the worst offenders are represented by only 16 distinct chemical substructures, originally termed Family_Filter_A chemotypes [10]. Second-generation–assay platform-independent filters more recently developed by the pharmaceutical industry [17] and academic researchers [18] have validated the problem imposed by this subset of 16 structures alone.

**Table 1:** Filters for PAINS and promiscuous compounds.

Filter	URL	Description	Input Format
FAFdrugs4	http://fafdrugs4.mti.univ-paris-diderot.fr	Predicts ADME parameters’ physicochemical characteristics, PAINS filtering using predefined or custom filtering parameters. Optional use of Eli Lilly and original PAINS compendium [6].	SDF
ZINC	http://zinc15.docking.org/patterns/home	PAINS and aggregate filter. PAINS definitions based on the original compendium [6].	SMILES, ZINC ID, InChl, SMARTS
SwissADME	swissadme.ch	Calculates physicochemical characteristics, drug-like properties, pharmacokinetic properties, and PAINS filtering and predicts ADME parameters. PAINS definition based on the original compendium [6].	SMILES
PAINS-Remover	http://www.cbligand.org/PAINS/	PAINS filter; definitions based on the original compendium [6].	SMILES
Knime	https://www.knime.com/forum/indigo/pains-filter-workflow	Online workflow for PAINS filtering; definitions based on the original compendium [6].	SMARTS
Badapple	http://pasilla.health.unm.edu/tomcat/badapple/badapple	Second-generation filter algorithm, moving beyond substructure searches to identify potential promiscuous compounds from database of known scaffolds. Uses the Molecular Libraries Small Molecule Repository (MLSMR) database of compounds and PubChem HTS assays. Total structure library is approximately 390,692 compounds [18].	SMILES
Aggregator Advisor	advisor.bkslab.org	Identifies molecules that may or have been shown to aggregate in biochemical assays. Based on physical properties and chemical similarity to known aggregators.	SMILES

Note the potential for translation issues from the original SLN PAINS filter language into SMARTS [10]. **Abbreviations**: ADME, absorption, distribution, metabolism, and excretion; HTS, high-throughput screening; InChI, international chemical identifier; MLSMR, Molecular Libraries Small Molecule Repository; PAINS, pan-assay interference compounds; SDF, structure-data file; SLN, SYBYL line notation; SMARTS, SMILES arbitrary target specification; SMILES, simplified molecular-input line-entry system.

## Why does it matter?

Realizing that a coveted hit candidate is a promiscuous nuisance is upsetting but without negative impact on the field at large. However, PAINS and IMPs have contaminated the scientific literature in the form of thousands of published articles. The concern is usually not that individual experiments cannot be reproduced—quite the opposite, in fact—but that mechanistic models, claims of target selectivity, and/or excited predictions concerning translational potential are almost always unsubstantiated [5]. This avalanche of publications has created a self-perpetuating system, since commercial vendors often add compounds containing PAIN functionalities to their catalogues as supposedly selective bioactives, and later studies build on the earlier reports.

In addition to intense debate, the problem has triggered severe countermeasures in the medicinal chemistry field. In 2017, eight American Chemical Society (ACS) journals implemented a joint policy that newly discovered bioactives must be examined for known undesirable chemotypes, activity claims be supported by appropriate experimental strategies, and the specific activity of flagged compounds be validated by at least two different assays [8].

**Fig 1:**
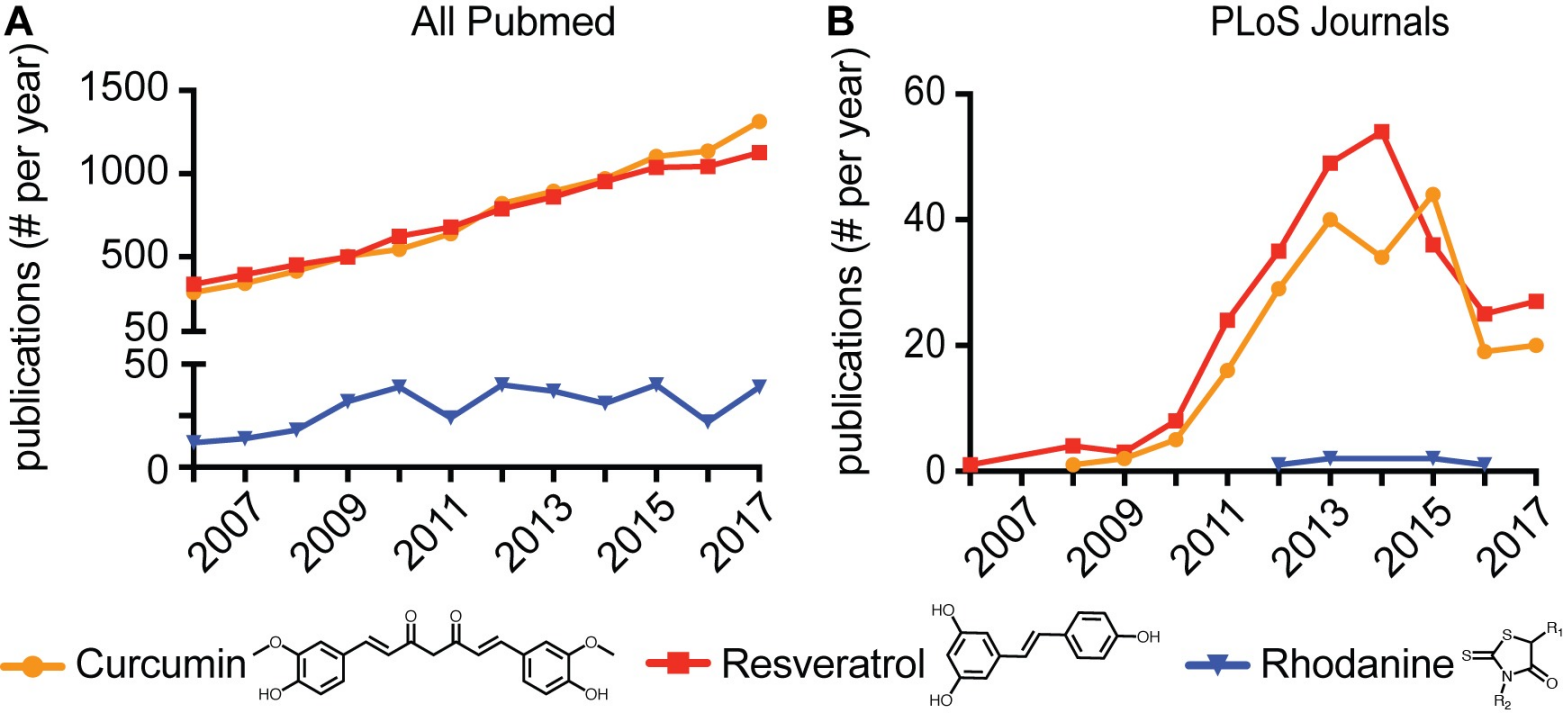
Number of publications per year since 2006 associating bioactivities with a subset of three PAINS: Rhodanine analogs, curcumins, and resveratrol. A) All PubMed-indexed journals. B) All PLOS journals. Searches were conducted using “compound name” and publication years 2006–2017 as keywords and for (B) triaged for articles associating bioactivities with these compounds.

Although biologists and biology journals are active contributors to drug discovery, an equivalent tackling of the problem is lacking. For instance, among the worst individual PAINS chemotypes are rhodanine analogs [6] and polyhydroxylated natural phytochemical IMPs such as curcumin and resveratrol [5, 19]. Rhodanines have shown a stable presence in the literature, and both of these IMPs enjoyed a steady increase in paper number per year in the past decade (Fig 1A). Limiting this survey to PLOS journals, we found a comparable rhodanines profile and, encouragingly, a decline in the appearance of both IMPs since 2014–2015 (Fig 1B). However, both have since plateaued at midlevel, indicating that the fight against PAINS may have started in the PLOS landscape, but the endgame of eradication must still be won.

## Reasons for the persistence of PAINS and IMPs in the biology literature

Better herd immunity among biologists against publishing undesirable chemical scaffolds is therefore needed, which will require addressing the root cause of the problem. We believe that the reasons for persistence of PAINS in the biological literature are 3-fold: poor curation of many preassembled chemical libraries, inexperience of many biologists in medicinal chemistry, and an unwillingness to drop a compromised scaffold despite strong experimental evidence for major liabilities.

Older diversity sets in particular are insufficiently curated against known problem chemotypes, returning a high hit rate in HTS campaigns that fails to deliver viable candidates. This problem extends to the Library of Pharmacologically Active Compounds (LOPAC) and compendiums of approved drugs that are revisited in the popular framework of drug-repurposing screens. Many bioactives originated from discontinued drug development campaigns, sometimes effectively creating PAIN-enriched collections, and approximately 5% of licensed drugs contain PAIN moieties [6, 10, 20]. These PAIN drugs were never subjected to the rigorous triaging system of modern drug development. For instance, doxorubicin—a quinone analog—appeared as a hit in every antiviral screen that we have conducted against a bioactives library and was active in approximately 85% of over 4,000 published assays [14, 20].

Biologists new to the chemical biology field are at greatest risk of selecting PAINS as screening “hits” based on their tantalizing initial bioactivities, often launching elaborate characterization and development studies. As time and major resources are invested, the desire to publish often prevails, even if it becomes obvious that no viable path to the clinic exists. In this case, the study justification usually switches to application of the identified compound as a useful chemical probe in future work. Quite the opposite is true, however. A probe compound must be more target selective and mechanistically precise than an actual drug to be of value [10], eliminating meaningful applications of PAINS and IMPs as research tools.

## Basic measures to halt perpetuation of the problem

In order to overcome undesirable chemical scaffolds, a joint effort of biology researchers, reviewers, and editors will be required, matching that underway in the medicinal chemistry field. In the case of publishing bioactive chemicals in biology journals, however, some very basic reporting standards are lacking that must be established. We suggest the mandatory inclusion of four checkpoints into the submission package of studies investigating bioactivities of small-molecule chemicals:

1. Basic compound information: To expedite the cross-referencing of compounds against chemical databases and filter algorithms, structures must be submitted in electronically readable formats such as the simplified molecular-input line-entry system (SMILES) or molecular formula strings. In many biology journals, compounds are still described by two-dimensional structure drawings only, making it unnecessarily cumbersome to integrate structures with chemical software. To eliminate the publication of nonreproducible bioactivities that are due, for instance, to compound decomposition or chemical impurities, hits must furthermore be synthetically validated, and substance purity stated.
2. Electronic filtering of undesirable chemotypes: A number of filter algorithms are publicly available that provide a first-pass view of the overall drug likeness of screening hits (Table 1). We have summarized the ongoing discussion of limitations of these filters, but major structural liabilities are recognized with sufficient accuracy to provide valuable information for reviewers and editors. We propose that submissions of new scaffolds for publication should be accompanied by analysis reports from at least two algorithms. Strategies to support development and maintenance of these filter servers should in parallel be discussed on an interdisciplinary level to ensure long-term public access.
3. Activity searches against chemical databases: Summaries of substructure searches of chemical databases such as SciFinder or PubChem against a submitted scaffold need to be provided to facilitate detection of potential frequent hitters. Although drugs can display polypharmacological behavior, and a diverse set of biological effects is therefore not a knock-out criterion [14], scaffolds associated with more than one supposed target must be treated with caution.
4. Experimental validation: Publication of an identified Filter_A PAIN [10] needs to be justified by a detailed experimental characterization supporting specificity of the reported activity. For instance, positive target identification through characterization of direct compound binding using label-free technologies such as surface-plasmon resonance, biolayer interferometry, or isothermal titration calorimetry; the generation of informative resistance profiles for target characterization of pathogen-directed antimicrobials; and the development of a meaningful synthetic structure-activity relationship should be requested for a hit with severe potential liabilities.

In our opinion, implementation of these checkpoints is urgently needed to ensure the credibility of drug-discovery studies published in biological journals. Encouraged by the changes established by the ACS, we are optimistic that we can regain focus on exciting and developable drug candidates.

## References

[1] Medicines Use and Spending in the U.S. A Review of 2016 and Outlook to 2021. [Internet]. IQVIA Institutue for Human Data Science. 2017 [cited 03/16/2018]. Available from: https://www.iqvia.com/institute/reports/medicines-use-and-spending-in-the-us-a-review-of-2016.

[2] Thorne N, Auld DS, Inglese J. Apparent activity in high-throughput screening: origins of compound-dependent assay interference. Curr Opin Chem Biol. 2010;14(3):315–24. Epub 2010/04/27. doi: 10.1016/j.cbpa.2010.03.020 ; PubMed Central PMCID: PMCPMC2878863. 20417149 10.1016/j.cbpa.2010.03.020 PMC2878863

[3] Dahlin JL, Inglese J, Walters MA. Mitigating risk in academic preclinical drug discovery. Nat Rev Drug Discov. 2015;14(4):279–94. Epub 2015/04/02. doi: 10.1038/nrd4578 . 25829283 10.1038/nrd4578 PMC6002840

[4] Thorne N, Shen M, Lea WA, Simeonov A, Lovell S, Auld DS, et al Firefly luciferase in chemical biology: a compendium of inhibitors, mechanistic evaluation of chemotypes, and suggested use as a reporter. Chem Biol. 2012;19(8):1060–72. Epub 2012/08/28. doi: 10.1016/j.chembiol.2012.07.015 ; PubMed Central PMCID: PMCPMC3449281. 22921073 10.1016/j.chembiol.2012.07.015 PMC3449281

[5] Baell J, Walters MA. Chemistry: Chemical con artists foil drug discovery. Nature. 2014;513(7519):481–3. doi: 10.1038/513481a . 25254460 10.1038/513481a

[6] Baell JB, Holloway GA. New substructure filters for removal of pan assay interference compounds (PAINS) from screening libraries and for their exclusion in bioassays. J Med Chem. 2010;53(7):2719–40. doi: 10.1021/jm901137j . 20131845 10.1021/jm901137j

[7] Bisson J, McAlpine JB, Friesen JB, Chen SN, Graham J, Pauli GF. Can Invalid Bioactives Undermine Natural Product-Based Drug Discovery? J Med Chem. 2016;59(5):1671–90. Epub 2015/10/28. doi: 10.1021/acs.jmedchem.5b01009 ; PubMed Central PMCID: PMCPMC4791574. 26505758 10.1021/acs.jmedchem.5b01009 PMC4791574

[8] Aldrich C, Bertozzi C, Georg GI, Kiessling L, Lindsley C, Liotta D, et al The Ecstasy and Agony of Assay Interference Compounds. ACS Cent Sci. 2017;3(3):143–7. Epub 2017/04/08. doi: 10.1021/acscentsci.7b00069 ; PubMed Central PMCID: PMCPMC5364449. 28386587 10.1021/acscentsci.7b00069 PMC5364449

[9] Rishton GM. Nonleadlikeness and leadlikeness in biochemical screening. Drug Discov Today. 2003;8(2):86–96. Epub 2003/02/05. . 12565011 10.1016/s1359644602025722

[10] Baell JB, Nissink JWM. Seven Year Itch: Pan-Assay Interference Compounds (PAINS) in 2017-Utility and Limitations. ACS Chem Biol. 2018;13(1):36–44. Epub 2017/12/05. doi: 10.1021/acschembio.7b00903 ; PubMed Central PMCID: PMCPMC5778390. 29202222 10.1021/acschembio.7b00903 PMC5778390

[11] Ingolfsson HI, Thakur P, Herold KF, Hobart EA, Ramsey NB, Periole X, et al Phytochemicals perturb membranes and promiscuously alter protein function. ACS Chem Biol. 2014;9(8):1788–98. Epub 2014/06/06. doi: 10.1021/cb500086e ; PubMed Central PMCID: PMCPMC4136704. 24901212 10.1021/cb500086e PMC4136704

[12] Erlanson DA. Learning from PAINful lessons. J Med Chem. 2015;58(5):2088–90. Epub 2015/02/25. doi: 10.1021/acs.jmedchem.5b00294 . 25710486 10.1021/acs.jmedchem.5b00294

[13] Dahlin JL, Nissink JW, Strasser JM, Francis S, Higgins L, Zhou H, et al PAINS in the assay: chemical mechanisms of assay interference and promiscuous enzymatic inhibition observed during a sulfhydryl-scavenging HTS. J Med Chem. 2015;58(5):2091–113. Epub 2015/01/31. doi: 10.1021/jm5019093 ; PubMed Central PMCID: PMCPMC4360378. 25634295 10.1021/jm5019093 PMC4360378

[14] Capuzzi SJ, Muratov EN, Tropsha A. Phantom PAINS: Problems with the Utility of Alerts for Pan-Assay INterference CompoundS. J Chem Inf Model. 2017;57(3):417–27. Epub 2017/02/07. doi: 10.1021/acs.jcim.6b00465 ; PubMed Central PMCID: PMCPMC5411023. 28165734 10.1021/acs.jcim.6b00465 PMC5411023

[15] Senger MR, Fraga CA, Dantas RF, Silva FP Jr. Filtering promiscuous compounds in early drug discovery: is it a good idea? Drug Discov Today. 2016;21(6):868–72. Epub 2016/02/18. doi: 10.1016/j.drudis.2016.02.004 . 26880580 10.1016/j.drudis.2016.02.004

[16] Chai CL, Matyus P. One size does not fit all: Challenging some dogmas and taboos in drug discovery. Future Med Chem. 2016;8(1):29–38. Epub 2015/12/23. doi: 10.4155/fmc.15.167 . 26689236 10.4155/fmc.15.167

[17] Nissink JWM, Blackburn S. Quantification of frequent-hitter behavior based on historical high-throughput screening data. Future Med Chem. 2014;6(10):1113–26. doi: 10.4155/fmc.14.72 PubMed PMID: WOS:000346694000007. 25078133 10.4155/fmc.14.72

[18] Yang JJ, Ursu O, Lipinski CA, Sklar LA, Oprea TI, Bologa CG. Badapple: promiscuity patterns from noisy evidence. J Cheminform. 2016;8:29 Epub 2016/05/31. doi: 10.1186/s13321-016-0137-3 ; PubMed Central PMCID: PMCPMC4884375. 27239230 10.1186/s13321-016-0137-3 PMC4884375

[19] Nelson KM, Dahlin JL, Bisson J, Graham J, Pauli GF, Walters MA. The Essential Medicinal Chemistry of Curcumin. J Med Chem. 2017;60(5):1620–37. Epub 2017/01/12. doi: 10.1021/acs.jmedchem.6b00975 ; PubMed Central PMCID: PMCPMC5346970. 28074653 10.1021/acs.jmedchem.6b00975 PMC5346970

[20] Baell JB. Feeling Nature's PAINS: Natural Products, Natural Product Drugs, and Pan Assay Interference Compounds (PAINS). J Nat Prod. 2016;79(3):616–28. Epub 2016/02/24. doi: 10.1021/acs.jnatprod.5b00947 . 26900761 10.1021/acs.jnatprod.5b00947

